# Preferential activation of small cutaneous fibers through small pin electrode also depends on the shape of a long duration electrical current

**DOI:** 10.1186/s12868-019-0530-8

**Published:** 2019-09-14

**Authors:** Rosa Hugosdottir, Carsten Dahl Mørch, Ole Kæseler Andersen, Thordur Helgason, Lars Arendt-Nielsen

**Affiliations:** 1 Center of Neuroplasticity and Pain, SMI®, Department of Health Science and Technology, Aalborg University, Fredrik Bajers Vej 7D3, 9220 Aalborg, Denmark; 2 Institute of Biomedical and Neural Engineering, Health Technology Center, School of Engineering and Science, Reykjavik University, Menntavegur 1, 101 Reykjavik, Iceland

**Keywords:** Electrical stimulation, Electrical pulse shapes, Pin electrode, Patch electrode, Small fiber activation, Large fiber accommodation

## Abstract

**Background:**

Electrical stimulation is widely used in experimental pain research but it lacks selectivity towards small nociceptive fibers. When using standard surface patch electrodes and rectangular pulses, large fibers are activated at a lower threshold than small fibers. Pin electrodes have been designed for overcoming this problem by providing a higher current density in the upper epidermis where the small nociceptive fibers mainly terminate. At perception threshold level, pin electrode stimuli are rather selectively activating small nerve fibers and are perceived as painful, but for high current intensity, which is usually needed to evoke sufficient pain levels, large fibers are likely co-activated. Long duration current has been shown to elevate the threshold of large fibers by the mechanism of accommodation. However, it remains unclear whether the mechanism of accommodation in large fibers can be utilized to activate small fibers even more selectively by combining pin electrode stimulation with a long duration pulse.

**Results:**

In this study, perception thresholds were determined for a patch- and a pin electrode for different pulse shapes of long duration. The perception threshold ratio between the two different electrodes was calculated to estimate the ability of the pulse shapes to preferentially activate small fibers. The perception threshold ratios were compared between stimulation pulses of 5- and 50 ms durations and shapes of: exponential increase, linear increase, bounded exponential, and rectangular. Qualitative pain perception was evaluated for all pulse shapes delivered at 10 times perception threshold. The results showed a higher perception threshold ratio for long duration 50 ms pulses than for 5 ms pulses. The highest perception threshold ratio was found for the 50 ms, bounded exponential pulse shape. Results furthermore revealed different strength-duration relation between the bounded exponential- and rectangular pulse shapes. Pin electrode stimulation at high intensity was mainly described as “stabbing”, “shooting”, and “sharp”.

**Conclusion:**

These results indicate that long duration pulses with a bounded exponential increase preferentially activate the small nociceptive fibers with a pin electrode and concurrently cause elevated threshold of large non-nociceptive fibers with patch electrodes.

## Background

Using electrical stimulation in human pain research is attractive as it has a potential to probe the nociceptive system with a quantifiable, safe, and reproducible stimulus [1]. Its main disadvantage is the lack of selectivity for activating small-diameter fibers, which is problematic in human experimental pain studies, as the nociceptive Aδ and C fibers have smaller diameter than the non-nociceptive Aβ fibers. A more preferential small-diameter fiber activation could be beneficially applied in experimental pain studies investigating long-lasting hyperalgesia [2–4] and membrane properties in cutaneous nerve fibers [5]. The small diameter (Aδ and C fibers) and the large diameter Aβ fibers will in this paper be referred to as small and large fibers, respectively.

For rectangular pulse shapes delivered with standard patch electrodes, large fibers have a lower activation threshold due to their large inter-nodal spacing and fiber diameter [6]. This combination of stimulation parameters and electrodes is typically used in transcutaneous electrical nerve stimulation to alleviate pain by activation of the large fibers [7]. This is contrary to the desired excitation of small fibers in experimental pain studies where co-activation of large fibers will interfere with pain processing and thereby complicate the interpretation of experimental pain results.

Intra-epidermal stimulation [1] and stimulation with cutaneous pin electrodes [2, 8] are methods to stimulate with high current density in the upper layer of the skin, where primarily small fibers terminate. At low intensity, the electrical field spreads closer to the small fibers, which favors their activation [6, 9], but co-activation of large fibers is likely at higher intensities [9]. Selectivity towards small fibers is further supported as evoked potential latencies have been shown to match those of Aδ fibers [1, 9, 10] and painful sensations, described as sharp and pricking, have been reported to pin electrode stimulation with intensities close to perception threshold (PT) [1, 2].

Additionally, different pulse shapes have been proposed for reversing the activation order of the fibers. Short duration (2 ms) linearly increasing pulses [11] and exponentially increasing pulses [12] have been reported to preferentially activate small fibers. Furthermore, sub-threshold pre-pulses [6, 13] and slowly rising pulses [12, 14] are methods that utilize the non-linear conduction properties of the fiber membrane to alter the nerve fiber excitability. Accommodation occurs when a nerve fiber is depolarized by slowly rising current (ramp depolarization) [15]. The result appears as an increased threshold for generating an action potential [15, 16], which affects the large fibers prior to the small fibers [6]. Accommodation has to the authors’ knowledge not been investigated for the pin electrode and it still remains unknown whether using a pin electrode in combination with a long duration current can be utilized for preferential activation of small fibers in human experimental settings.

In this study, it was assumed that large- and small nerve fibers were activated with patch- and pin electrodes, respectively. For each pulse shape and electrode combination, the subjective PT was found as indication of the fiber activation. The aim of this study was to evaluate the ability of different pulse shapes to cause a large PT ratio between these electrodes and thereby optimize the small fiber activation.

## Methods

### Subjects

The experiment was performed at Reykjavik University, Iceland and included 25 healthy individuals (12 females and 13 males, 18–67 years; mean age 35.5). Data from one subject was excluded from analysis due to a very high PT measurement (> 10 times interquartile range). The study was performed according to the declaration of Helsinki and study approval was obtained from the Icelandic national bioethics committee (Vísindasiðanefnd, Approval Number: 14-150). The subjects gave their written informed consent to participation in the experiment. Only healthy individuals were included in this experiment and exclusion criteria were pregnancy or breast feeding, addiction to cannabis, -opioids or -other drugs, skin diseases, past history of conditions possibly leading to neuropathy, and pain-relieving medication within the last 48 h.

### Experimental setup

Subjects were seated comfortably in a chair with arms resting on a pillow. Electrical stimulation was delivered by a voltage controlled electrical current stimulator (DS5; Digitimer Ltd; Welwyn Garden City, UK) on the volar forearm of the subjects. A personal computer with a custom made-program (LabVIEW, National Instruments) and a data acquisition card (USB-6351; National Instruments) were used for controlling the current stimulator.

**Fig. 1:**
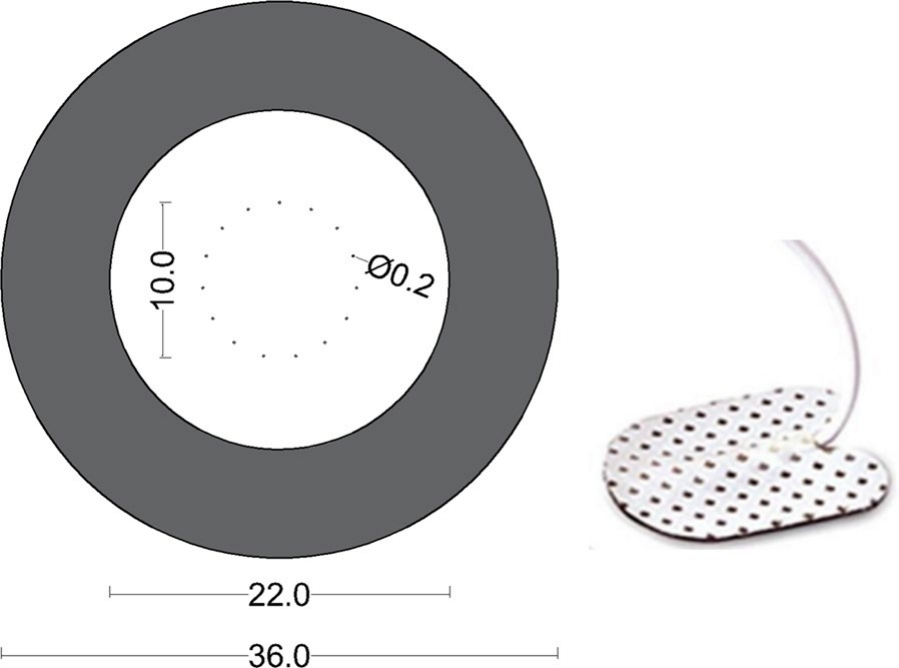
Electrodes applied in the experiment. Dimensions are shown in mm. Left) pin electrode is composed of 15 small diameter cathodes placed in a circle and a concentric anode. Right) patch electrode cathode

Two types of stimulation electrodes were applied (Fig. 1). A non-invasive pin electrode [10] and a standard surface patch electrode were applied for activating mainly small nociceptive- and large non-nociceptive fibers, respectively. The pin electrode was carefully cleaned with alcohol swaps between participants. Electrode type positioning on the left and right arm was randomized between subjects. The pin electrode cathode consisted of 15 small diameter (0.2 mm) blunted stainless steel pin array placed in circle with a diameter of 10 mm. The anode consisted of a concentric stainless steel ring, with an inner diameter of 22 mm and an outer diameter of 36 mm. The patch cathode was a 15 mm × 20 mm Ag–AgCl electrode (Neuroline 700, Ambu A/S, Ballerup, Denmark) with an anode of 5 cm × 9 cm (Pals Neurostimulation electrode, Axelgaard, Co., Ltd., Fallbrook, CA, USA). The cathodes of the pin- and patch electrodes were placed 5 cm distal to the cubital fossa. The patch electrode anode was placed on the dorsal forearm.

### Pulse shapes

Four pulse shapes were applied including three different slowly rising currents and a rectangular pulse (see Table 1):

**Table 1:** Mathematical expressions and labels of the pulse shapes applied in the experiment. The pulse shapes originating from the provided equations are illustrated

Pulse shape	Equations	Label	Illustration
Exponential increase	$$i\left( t \right) = \left\{ {\begin{array}{*{20}l} {\frac{{I_{s} }}{{e^{{\frac{{T_{S} }}{\tau }}} - 1}}\left( {e^{{\frac{t}{\tau }}} - 1} \right),\quad 0 \le t < Ts } \\ {I_{s} \cdot e^{{\frac{ - t}{{\tau_{tr} }}}} , \qquad \qquad T_{s} \le t \le T_{tr} } \\ \end{array} } \right.$$	Exp	
Linear increase	$$i\left( t \right) = \left\{ {\begin{array}{*{20}l} { \frac{{I_{s} }}{{T_{s} }} \cdot t, \qquad 0 \le t < Ts } \\ {I_{s} \cdot e^{{\frac{ - t}{{\tau_{tr} }}}} , \quad T_{s} \le t \le T_{tr} } \\ \end{array} } \right.$$	Lin	
Bounded exponential	$$i\left( t \right) = \left\{ {\begin{array}{*{20}l} { \frac{{I_{s} }}{{1 - e^{{\frac{{ - T_{S} }}{\tau }}} }}\left( {1 - e^{{\frac{ - t}{\tau }}} } \right), \quad 0 \le t < Ts} \\ {I_{s} \cdot e^{{\frac{ - t}{{\tau_{tr} }}}} , \,\,\,\, \qquad \qquad T_{s} \le t \le T_{tr} } \\ \end{array} } \right.$$	B.Exp	
Rectangular	$i\left( t \right) = I_{s} \left( t \right), \quad 0 \le t < Ts$	Rec	

$I_{s}$  stimulation current, $T_{S}$  stimulation duration, $\tau$  ($T_{S}$ /2)  time constant. Trailing phase: $T_{tr}$  $T_{S} *$ 1.4 and $\tau_{tr}$  $\tau$ /6.6

1. Exponential increase (Exp).
2. Linear increase (Lin).
3. Bounded exponential (B.Exp).
4. Standard rectangular (Rec).

An exponential trailing phase was added to slowly rising pulse shapes to avoid anodal break excitation [12, 17]. Three durations of the Rec pulse shape were applied (1 ms, 5 ms, and 50 ms) whereas two duration (5 ms and 50 ms) of slowly rising pulse shapes were applied. The mathematical expressions, labels, and illustrations of the pulse shapes are presented in Table 1. Moreover, charges for specific pulse shapes and durations were calculated for an exemplified current intensity of 1 mA (Table 2). The total charge was found by integrating the pulse shape function (shown in Table 1) over the entire pulse duration. The pulses generated by the stimulator, while stimulating over a resistor and capacitor in parallel, were measured on an oscilloscope to verify the described parameters.

**Table 2:** The pulse durations applied in the experiment and an exemplified charge calculated for a peak intensity of 1 mA

Pulse form	Rec			Exp		Lin		B.Exp	
Duration (ms)	1	5	50	5	50	5	50	5	50
Charge (µC)	1	5	50	2.09	20.94	2.88	28.77	3.66	36.59

### Data collection

#### Subjective electrical perception thresholds

The PT of the patch- and pin electrode, which indicated activation of large and small fibers, respectively, was determined for all pulse shapes in randomized order for both electrodes. The PTs were determined using the method of limits which included three repetition of the following staircase procedure: Starting at sub-PT intensity, single pulses were delivered at 0.5 Hz and the intensity of each pulse was increased with a factor of 1.05 until the subject perceived the stimulation pulses and pressed a handheld custom-made button (Reykjavik University). The intensity was then increased to 110% of the last stimulation and single pulses were again delivered at 0.5 Hz with a decreasing factor of 0.95 until the subject pressed the button, indicating that the perception had dissipated. Before repeating the procedure, the stimulation intensity was automatically decreased to 90% of the last stimulation. The PT was determined as the average of the six intensities at which the subject had pressed the button. Before identifying the PT, a training sequence of this staircase procedure was carried out in order to familiarize the subjects with the sensation of the low current electrical pulses.

#### Qualitative measure of pain perception

For both electrodes and each pulse shape, a single stimulation pulse with a current intensity of 10 times PT was delivered after identification of each PT. The subjects were subsequently asked to describe the qualitative pain sensation to these stimuli by using the short form of the McGill pain questionnaire (SF-MPQ), English version [18]. The SF-MPQ consisted of sensory and affective pain descriptors with an intensity scale (0 = none, 1 = mild, 2 = moderate, 3 = severe) and a visual analogue scale (VAS) were pain was rated from 0 (no pain) to 10 (worst pain imaginable). The descriptors were explained to the subjects prior to the experiment if needed.

### Data analysis

#### Subjective electrical perception thresholds

The currents applied at PT (IPT) were analyzed using a three-way repeated measures analysis of variance (RM-ANOVA) with electrode (pin- and patch electrode), pulse duration (5-, and 50 ms) and pulse shape (Exp, Lin, B.Exp and Rec) as within-subject factors. In case of a three-way interaction, a two-way RM-ANOVA was used to analyze IPT for each electrode separately. As the pulse shapes deliver different amount of charge for the same current intensity, separate charge-based analysis was performed on the charge delivered to obtain the PT (QPT) to ensure that the differences between the IPT were not only observed to equal the amount of charge delivered by the different pulse shapes. The charge-based analysis was performed using the same statistical model as described for the IPT.

The PT-ratio between patch- and pin electrode was calculated as an estimate of the ability of the pulse shapes to preferentially activate nociceptive fibers. PT-ratios were analyzed using a two-way RM-ANOVA with duration and pulse shape as within-subject factors. Note that the PT-ratio is equal for IPT and QPT.

The strength-duration curves of both electrodes were analyzed by fitting the IPT for the three durations (1, 5-, and 50 ms) to Weiss Law [19] for comparison to earlier studies. The rheobase and chronaxie were estimated by least square estimation of strength-duration curves. The rheobases and chronaxies were compared between the two electrode types using Wilcoxon signed rank test in accordance with data distribution.

#### Pain perception and qualitative description

The intensity ratings of the quality descriptors were analyzed using a three-way RM-ANOVA with electrode (patch- and pin electrode), pulse shape with duration (1 ms Rec, 5 ms Exp, − Lin, − B.exp, − Rec, 50 ms Exp, − Lin, − B.Exp and − Rec), and descriptor (throbbing, shooting, stabbing, sharp, and cramping) as within-subject factors. The VAS rating was analyzed using a three-way RM-ANOVA with electrode, pulse shape, and duration as within-subject factors.

Greenhouse–Geisser was applied for correction of non-sphericity. Data for IPT, QPT, VAS, and qualitative measures are presented as mean ± standard error of mean (SEM) and data for rheobase and chronaxie are presented as medians and quartile (IQR). P values < 0.05 were considered as statistically significant. Post Hoc test with Sidak adjustment was used to adjust for multiple comparisons.

## Results

### Subjective electrical perception thresholds

**Fig. 2:**
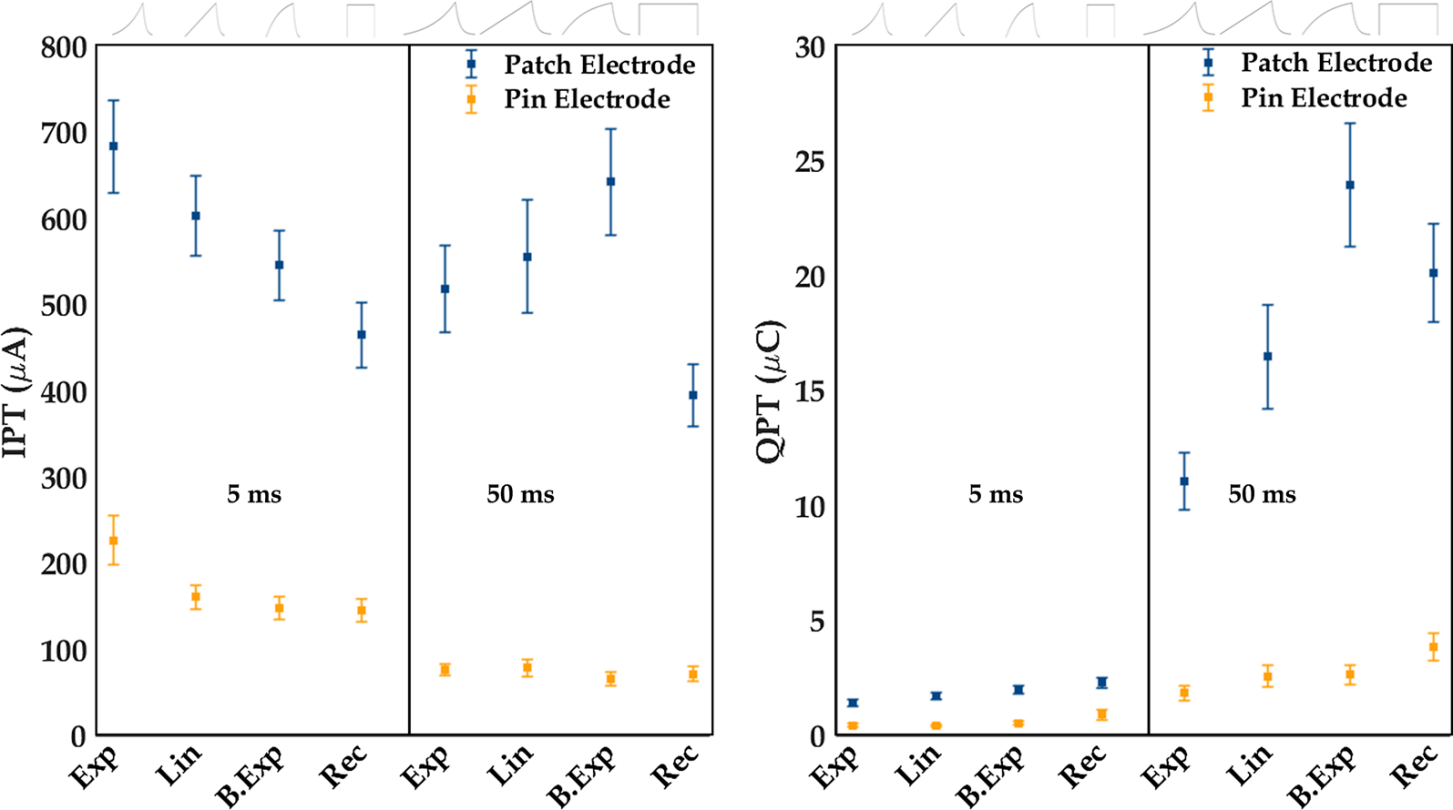
Perception thresholds are shown (mean ± standard error) as current (IPT) on the left and as charge (QPT) on the right. IPT and QPT are shown for all pulse forms of 5- and 50 ms durations for the pin- and the patch electrodes. *Exp* exponential increase, *Lin* linear increase, *B.Exp* bounded exponential, *Rec* rectangular. Blue line = patch electrode, orange line = pin electrode. The pulse shapes are illustrated above the figure

Analysis of the IPTs and QPTs (Fig. 2) revealed three-way interactions, between electrode, duration, and pulse shape (IPT: F(2.46,56.57) = 5.50, p = 0.004., QPT: F(3,72) = 24.02, p < 0.001). Two-way RM-ANOVAs for the pin electrode revealed significant interactions between pulse shapes and durations (IPT: F(1.60,36.75) = 3.90, p = 0.04, QPT: F(1.79,42.90) = 4.89, p = 0.02). For IPT, the interaction is explained by the IPT for 5 ms Exp, which was larger than the IPT of 5 ms Lin and Rec. As expected, the IPTs for the pin electrode were larger for the 5 ms durations than for the 50 ms durations (Table 3). The QPT was approximately constant for 5 ms pulses, but for 50 ms lowest QPT was observed for the Exp pulse shape. The two-way RM-ANOVAs for the patch electrode revealed interactions between the pulse shapes and durations (IPT: F(2.27,52.14) = 10.14, p < 0.001, QPT: F(2.37,56.90) = 29.33), p < 0.001). Post hoc comparison (Table 3) showed that for 5 ms pulses, the IPT of Exp, Lin and B.Exp were larger than the IPT of Rec and furthermore that the IPT of Exp and Lin were larger than the IPT of B.Exp. For 50 ms pulses, the IPT of Exp, Lin and, B. Exp pulses were larger than the IPT of Rec, and the IPT of B.Exp was also larger than the IPT of Exp pulses. The IPTs were larger for 5 ms duration than for 50 ms duration for Exp and Rec, whereas the IPTs were not different between the two durations for Lin and for B.Exp.

**Table 3:** Post hoc comparison with Sidak adjustment for two way RM-ANOVA analysis of the perception threshold for current (IPT) and charge (QPT)

Factor	Patch electrode		Pin electrode	
	Effect	*p* value	Effect	p-value
IPT				
Duration (5 ms)	Exp > B.Exp	< 0.001	Exp > Lin	0.049
	Exp > Rec	< 0.001	Exp > Rec	0.05
	Lin > B.Exp	0.042		
	Lin > Rec	< 0.001		
	B.Exp > Rec	< 0.001		
Duration (50 ms)	B.Exp > Exp	< 0.001	No differences	
	Exp > Rec	< 0.001		
	B.Exp > Rec	< 0.001		
	Lin > Rec	0.053		
Pulse shape (Exp)	5 > 50	< 0.001	5 > 50	< 0.001
Pulse shape (Lin)	5 = 50	0.408	5 > 50	< 0.001
Pulse shape (B.Exp)	5 = 50	0.066	5 > 50	< 0.001
Pulse shape (Rec)	5 > 50	0.009	5 > 50	< 0.001
QPT				
Duration (5 ms)	Exp < Lin	0.006	No differences	
	Exp < B.Exp	< 0.001		
	Exp < Rec	< 0.001		
	Lin < B.Exp	0.001		
	Lin < Rec	< 0.001		
	B.Exp < Rec	0.006		
Duration (50 ms)	Exp < Lin	0.003	Exp < B.Exp	0.029
	Exp < B.Exp	< 0.001	Exp < Rec	0.005
	Exp < Rec	< 0.001		
	Lin < B.Exp	< 0.001		
	B.Exp > Rec	0.017		
Pulse shape (Exp)	50 > 5	< 0.001	50 > 5	< 0.001
Pulse shape (Lin)	50 > 5	< 0.001	50 > 5	< 0.001
Pulse shape (B.Exp)	50 > 5	< 0.001	50 > 5	< 0.001
Pulse shape (Rec)	50 > 5	< 0.001	50 > 5	< 0.001

**Fig. 3:**
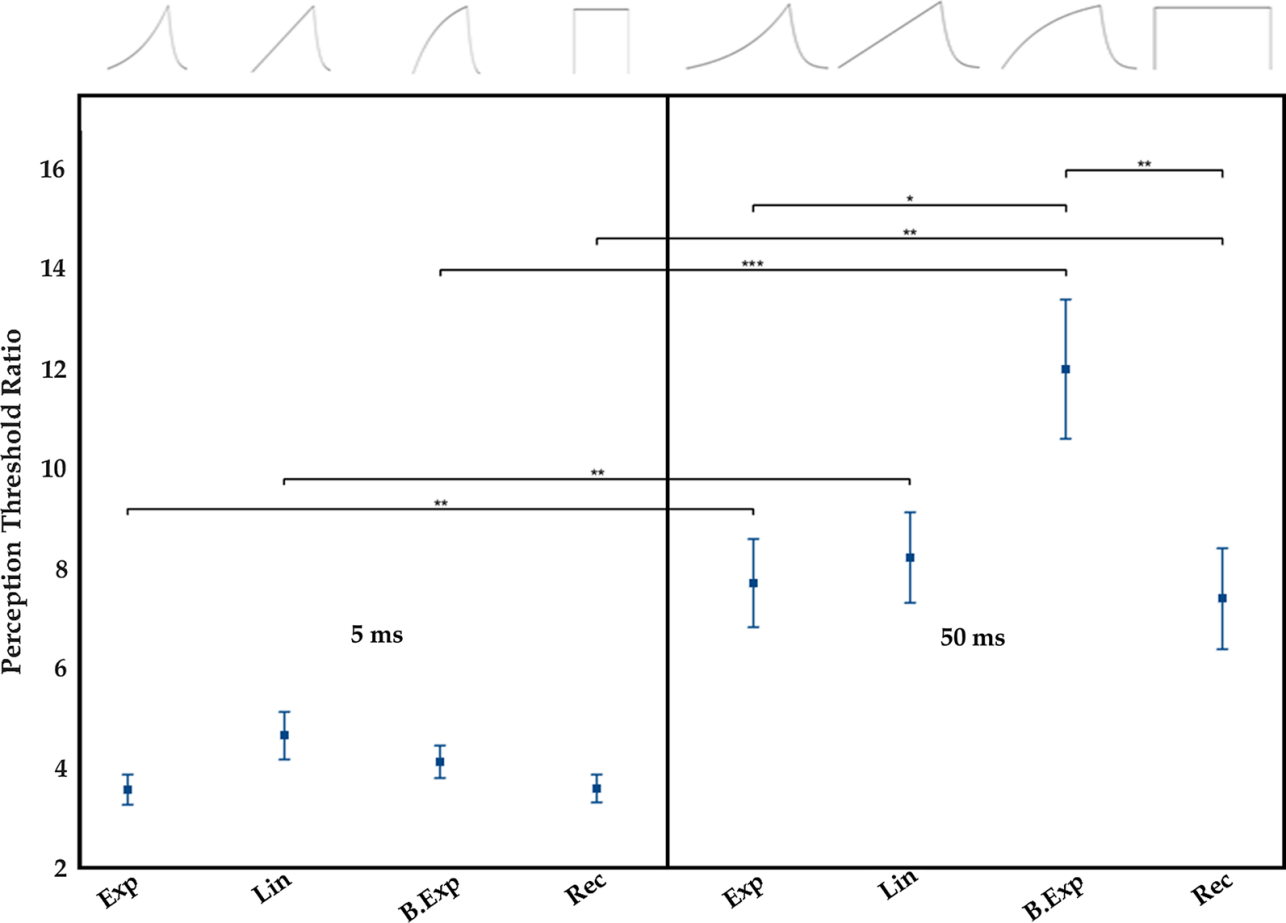
PT-ratios (mean ± standard error) between the patch- and the pin electrodes are shown for all pulse shapes and durations. *Exp* exponential increase, *Lin* linear increase, *B.Exp* bounded exponential, *Rec* rectangular. Statistical significant differences with Sidak multiple comparisons are included: *, **, and *** indicate p < 0.05, p < 0.01, and p < 0.001, respectively

For the patch electrode, the QPT increased as the shape of the pulse approached the rectangular pulse for all 5 ms pulses and also for 50 ms pulses, except for the B.Exp pulse shape, which QPT was larger than for the Rec pulse shape. Opposite to the analysis on IPT, QPT was larger for 50 ms than for 5 ms pulses for both electrodes. The PT-ratios between the patch and the pin electrodes are presented in Fig. 3. An interaction effect was found between duration and pulse shape F(3,69) = 4.70, p = 0.008. No differences were found between any of the PT-ratios of 5 ms pulse shapes, but for 50 ms pulse shapes, the largest PT-ratio was obtained for the B.Exp, which was significantly different from the PT-ratio of Exp and Rec but not from Lin (see Fig. 3).

The median of the estimated rheobase for pin electrode Rec pulses (0.06 mA, IQR = 0.05 mA) was significantly lower than for the patch electrode (0.40 mA, IQR = 0.31 mA, z = 4.09, p < 0.001). The median of the estimated chronaxie was significantly larger for the pin electrode (4.56 ms, IQR = 7.17 ms) than for the patch electrode (0.57 ms, IQR = 1.07 ms, z = − 3.26, p = 0.001, Wilcoxon signed rank test).

### Pain perception and qualitative description

**Fig. 4:**
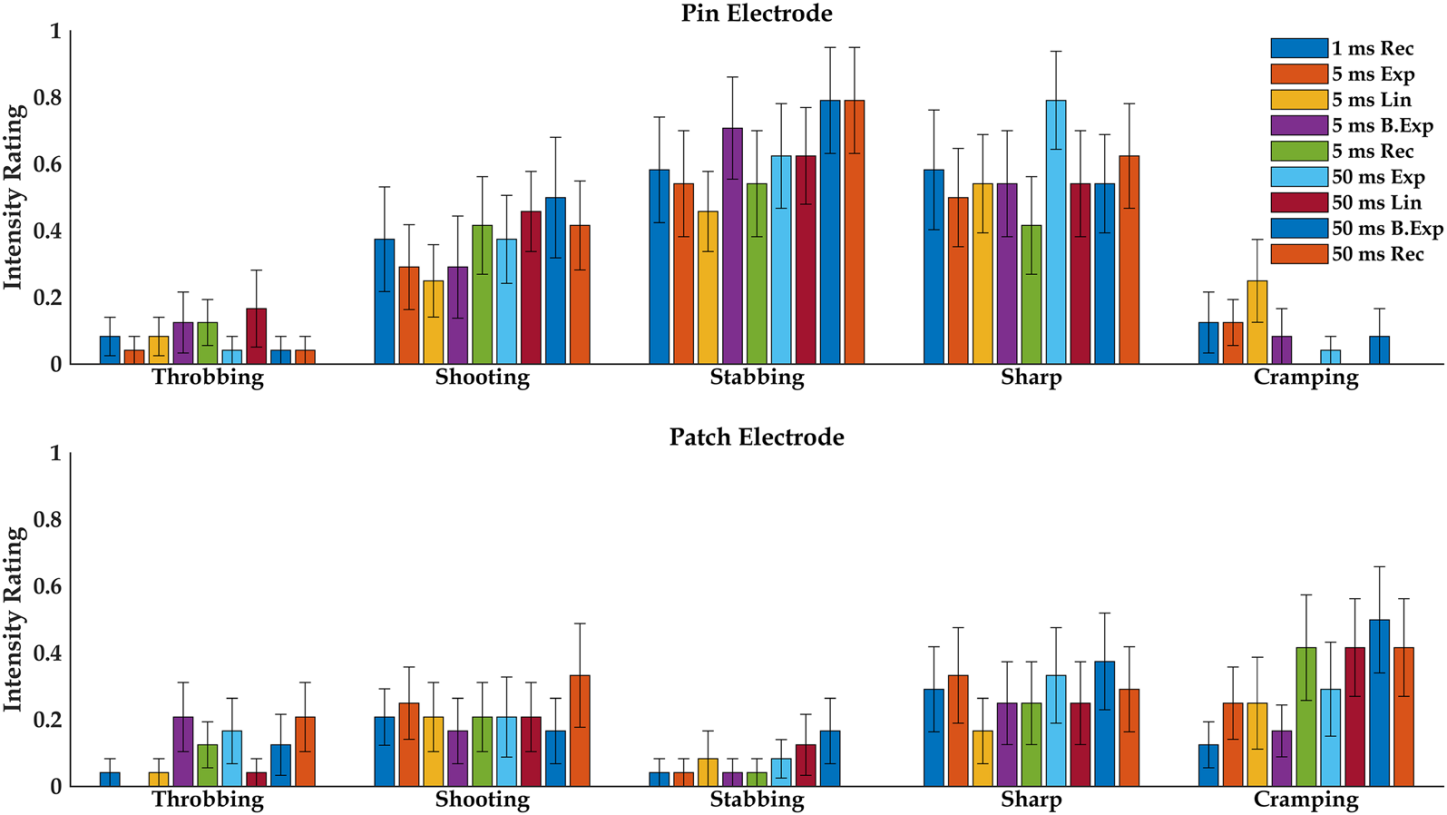
The individual intensities of each pain quality descriptor (mean ± standard error) for the pin electrode (upper graph) and the patch electrode (lower graph)

The perceived intensity for each quality descriptor averaged across subjects is presented for all pulse forms in Fig. 4. Quality descriptors with average intensity ratings below 0.2 for both electrodes were excluded (in total 10 out of 15 descriptors) from the analysis as these were only selected by minority of subjects and are therefore not believed to describe the main pain sensation felt by the subjects. There was no significant three-way interaction between quality descriptor, electrode and pulse shape F(9.67,222.33) = 1.68, p = 0.09. However, a two-way interaction was found between quality descriptor and electrode F(2.61,59.91) = 9.15, p < 0.001 but not between electrode and pulse shape F(5.01,115.14) = 0.61, p = 0.69, nor between pulse shape and descriptor F(7.44,171.17) = 0.74, p = 0.65. Multiple comparisons with Sidak adjustment are shown in Table 4.

**Table 4:** Post hoc comparison with Sidak adjustement for three-way RM ANOVA analysis of the qualitative pain ratings

Factor	Effect	p-value
Pain descriptor		
Shooting	Pin > patch	0.041
Stabbing	Pin > patch	< 0.001
Sharp	Pin > patch	0.041
Cramping	Patch > pin	0.013
Electrode		
Pin electrode	Stabbing > throbbing	< 0.001
Pin electrode	Stabbing > cramping	< 0.001
Pin electrode	Shooting > cramping	0.017
Pin electrode	Sharp > cramping	0.003
Pin electrode	Sharp > throbbing	0.031
Patch electrode	No differences	
Pulse shapes	No differences	

**Fig. 5:**
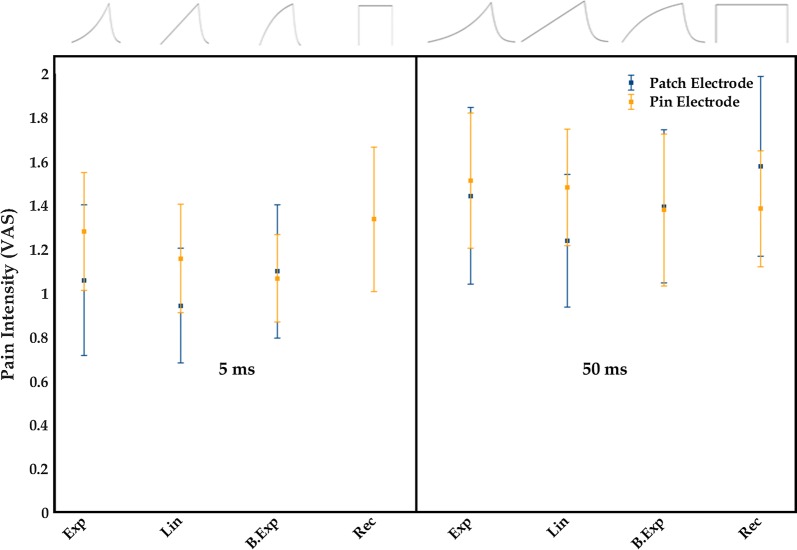
The pain intensity (VAS) (mean ± standard error) for stimulation of 10 times perception threshold is shown for the different pulse shapes and durations for the pin electrode and the patch electrode

The VAS pain ratings are shown in Fig. 5. Statistical analysis revealed a main effect of pulse duration, F(1,23) = 8.18, p = 0.009, which indicated that long duration pulses caused more pain than short duration pulses when PT was multiplied by 10. No significant interactions were observed and neither were main effects for electrodes and pulse shapes.

## Discussion

This study showed that the PT-ratio was in general larger for 50 ms- than for 5 ms duration and the largest PT-ratio was found for the 50 ms B.Exp pulse shape. This means that the 50 ms B.Exp pulse shape delivered via a pin electrode caused the lowest PT relative to the patch electrode PT and is therefore considered the optimal stimulation pulse form for preferential activation of small nociceptive fibers. Single pulses delivered with the pin electrode at high intensities normalized to 10 times each pulse shape’s PT were described as “stabbing”, “shooting”, and “sharp”, but no statistical differences were found between the pulse shapes. At high intensities, all of the pulse shapes likely activate small nociceptive fibers, but probably with the B.Exp pulse shape, co-activation of large non-nociceptive fibers is limited.

### Possible ion channel mechanisms during slowly increasing pulse shapes

Sub-threshold electrical currents can cause accommodation of nerve fibers by depolarizing the membrane [16]. The mechanism is dependent on the conduction properties of the fiber membrane, governed by different ion channels. Thus, the fast- and slow K_V_ channel [20, 21] and inactivation of Na_V_ channels [22] are believed to play a role in the accommodation phenomenon. The composition of the ion channels differs between the fiber types [23]. Na_V_ channel subtypes with different inactivation kinetics [24] (Na_V_ 1.8 and Na_V_ 1.9) are expressed to a greater extent on the small fibers than the large fibers [25–28], which may affect the fiber ability to accommodate to a depolarization. It has furthermore been proposed that the large fibers have a greater ability to accommodate than the small fibers [13]. The enlarged PT-ratio during long duration 50 ms pulses in the present study, and especially the B.Exp pulse shape, which is partly caused by an increase in patch electrode IPT is therefore most likely reflecting accommodation of large fibers. As accommodation seems not to be present when stimulating with the pin electrode, one hypothesis is that the slowly inactivating Na_V_ channel subtypes may be sensitive to long duration pulses and the B.Exp pulse may, with its initial sharp current increase, have a potential to activate the small fibers with the pin electrode. However for the patch electrode, the initial depolarization may not be enough to generate an action potential and towards the slowly increasing end of the pulse, a larger proportion of ion channels have been inactivated. This is supported by the analysis of QPT for the patch electrode, which revealed the largest QPT for B.Exp pulse, further indicating accommodation of large fibers.

### Comparing pulse shapes

Previous studies have applied different electrical pulse shapes to overcome the challenge of small fiber selectivity when using rectangular pulses. Sub-threshold pre-pulses have been suggested to reverse the activation order of large- and small fibers in animal settings [6, 29, 30] and similarly ramp pre-pulses with a subsequent rectangular test stimuli have been attempted for reversing the activation order of large- and small motor fibers in humans using conventional electrodes [13]. The study revealed an increased excitation threshold for linearly increasing pulses longer than 25 ms, which was more prominent for large than small fibers [13]. Data from the present study supports this and indicates that even more depolarization without neuronal firing could be caused by using the B.Exp pulse shape. Why the IPT of the Exp and Lin pulse shapes behave similar to the IPT of Rec pulses is not clear. One possible reason is that the effect of decreasing threshold caused by the strength-duration effect [31] and the effect of increasing threshold caused by accommodation cancels out between these two pulse durations. For the Exp pulse shape, it is possible that the very slowly rising current does not depolarize the nerve fiber sufficiently for opening Na_V_ channels. The fibers do therefore not accommodate sufficiently and the action potential is generated during the steep current increase at the end of the pulse, which causes similar strength-duration relationship as for the Rec pulse shape. The QPTs of Exp and Lin were furthermore smaller than for Rec pulses even though the IPTs were larger, which could indicate that the different IPTs were mainly observed to obtain equal charge across the pulse shapes. The differences in QPT may furthermore relate to the removal of charge from the nodes of ranvier prior to the initiation of an action potential, which could depend on both pulse duration and shape, since a larger charge was also observed for 50 ms than 5 ms pulses.

Studies investigating pre-pulses have shown that the threshold increase depends on the duration of the pre-pulse for both short duration rectangular pulses of 0.1–1 ms [29] and for long duration ramp pulses (50–500 ms) [13]. Furthermore, it has been shown that the increase in threshold is more pronounced when using pre-pulse intensities close to the excitation threshold [13, 29, 30]. Therefore the total charge delivered is likely not lower than using only one ramp pulse for stimulation. Limiting the charge is important for the safety of the participants as a large charge can cause acidification or even non-reversible damage to the tissue [32]. Therefore, single pulses that by themselves can activate small fibers preferentially were investigated in the present study. In a theoretical study, Hennings et al. [12] showed indication for an exponentially increasing pulse shape to reverse the activation order of large- and small motor nerves. Intra-epidermal stimulation has been suggested to activate Aδ- and C-fibers selectively by rectangular pulse stimulation (1 ms) or by using linear increasing pulses (2 ms) in several studies relying on evoked potential latencies [1, 9, 11, 33]. Inui and colleagues further proposed that long pulses with a non-steep current increase favor activation of C fibers [11]. This is in line with results from the present study, but longer pulse duration was needed for increasing the PT-ratio. Furthermore, slowly increasing currents presented as 4 Hz sine-wave stimulation, were recently shown to activate C-fibers preferentially in mice, pigs, and humans [34].

### Strength-duration curve

The PT is generally lower when stimulating with the pin electrode compared to stimulation with the patch electrode. This is a consequence of a relatively high current density when current is delivered through the small cathodal pins. When stimulating with rectangular pulses, the standard shape of the strength-duration curve has been replicated for both electrode types [5]; that is, a decrease in IPT with increased duration until a plateau is reached [31].

The rheobase and chronaxie were estimated by fitting the three IPTs for Rec pulses to Weiss law. The median of the estimated rheobase for stimulation through the pin- and the patch electrode was in line with results from a recent study where the pin- and patch electrode rheobase were reported at 0.07 ± 0.041 mA and 0.43 ± 0.10 mA, respectively [5]. The median of the estimated chronaxie was higher for the pin electrode, compared to the previous study where the chronaxie was estimated 1.060 ± 0.690 ms for the pin- and 0.580 ± 0.160 ms for the patch electrode [5]. One likely reason for the observed difference in chronaxie between the studies is that the shortest stimulation duration was 1 ms in the present study, which possibly caused an over-estimation of the chronaxie.

### Perception threshold as indication of fiber excitability

The current study uses the subjective PT as an indirect measure of the fiber excitability and the related limitations should be addressed. Before the electrical pulses are perceived, the signal travels from the primary afferent fibers through multiple neuronal synapses. The PT therefore also includes unknown cofounding factors (e.g. expectation and attention [35]), but these are likely similar for the different pulse shapes. Recently, the technique of perception threshold tracking was presented, where the patch- and the pin electrodes were used to assess membrane properties of large- and small fibers by use of subjective perception thresholds [5]. Similarly, perception thresholds to electrical stimulation of different frequencies have been related to activation of different fibers with the purpose of assessing peripheral neuropathy [36]. While identifying the PT for the different pulse shapes, single pulses were repeated at 0.5 Hz. During the session, a degree of habituation may have occurred, leading to higher PT towards the session end. The pulse shapes were delivered in a randomized order to account for potential habituation. Future directions on how to overcome the limitation of using the perception threshold as a measure of fiber excitability may include validation of the method by single trial electroencephalography recordings and neurophysiological recordings in animals. The qualitative description of subjective perception at the level of PT could also provide a dimension for discriminating between the activated fibers [10, 36, 37]. Robust assessment of perception quality at the level of PT is however hindered due to low perception intensity. This was even evident for high intensity stimulation of single pulses in current study. No differences were observed for VAS ratings between the electrodes nor pulse shapes and qualitative description did not differ between pulse shapes. This may be explained by floor effects, which were observed in ratings of qualitative descriptors and pain intensity (VAS). It should be mentioned that the stimulation intensity is higher when the patch electrode PT was multiplied by 10, and the current is therefore likely to have caused unpleasantness with the participants [38], which may have interfered with the pain ratings. The pin electrode stimulation however was described as more stabbing and sharp, supporting earlier findings regarding activation of small pain sensing fibers with pin electrode stimulation [38–40].

### Implications

The evidence of preferential small fiber activation using intra-epidermal and cutaneous stimulation with pin electrodes is mainly based on studies using current intensity close to perception threshold [8, 9]. In the present study, single pulse stimulation of painful intensity was applied for all pulse shapes in order to investigate the qualitative pain perception during the stimulation. For the pin electrode, quality of “stabbing”, “shooting” and “sharp” were selected which indicates activation of Aδ-fibers, however no difference in quality description was found between the pulse shapes. All of the pulse shapes may therefore at high intensity activate small nociceptive fibers but by applying long-duration pulses, co-activation of large fibers may be decreased. Utilizing the characteristics of the pin electrode or an intra-epidermal electrode to provide high current density at the small fiber endings and concurrently applying 50 ms B.Exp pulse to elevate the threshold of large non-nociceptive fibers could contribute as a valuable combination for achieving more preferential nociceptor activation. Methods using cutaneous electrical stimulation for inducing experimental long-lasting hyperalgesia, which have extensively been proposed in the literature [2–4] may benefit of using parameters for stimulation that are more selective for small nociceptive fibers.

## Conclusion

The results from this study showed that largest perception threshold-ratio between patch- and pin electrodes was obtained for long duration 50 ms bounded-exponential pulse shape, indicating optimal small fiber activation among the applied pulse shapes. The combination of the small pin electrode with bounded-exponential pulse shape provides high current density to the epidermis that favors small fiber activation in combination with accommodation of the large nerve fibers. The ability of electrical pulses to preferentially activate small fibers can be controlled by the stimulus pulse shape by utilizing different degrees of the large fiber accommodation. This effect may be exploited in human experimental pain studies.

## Data Availability

The datasets used and analyzed during the current study are available from the corresponding author on reasonable request.

## Abbreviations

PT: perception threshold
IPT: the current applied at PTs
QPT: the charge delivered to obtain the PT
Na_V_: voltage gated sodium channels
Exp: exponential increase
Lin: linear increase
B.Exp: bounded exponential
Rec: rectangular
SF-MPQ: short form McGill pain questionnaire
RM-ANOVA: repeated analysis of variance
SEM: standard error of mean
IQR: quartile
